# Resveratrol Enhances Temozolomide Efficacy in Glioblastoma Cells through Downregulated MGMT and Negative Regulators-Related STAT3 Inactivation

**DOI:** 10.3390/ijms24119453

**Published:** 2023-05-29

**Authors:** Moli Wu, Danyang Song, Hui Li, Nisar Ahmad, Hong Xu, Xiaobo Yang, Qian Wang, Xiaoxin Cheng, Sa Deng, Xiaohong Shu

**Affiliations:** 1 College of Pharmacy, Dalian Medical University, Dalian 116044, China; 2 College of Basic Medical Science, Dalian Medical University, Dalian 116044, China; 3 Key Laboratory for Basic and Applied Research on Pharmacodynamic Substances of Traditional Chinese Medicine of Liaoning Province, Dalian Medical University, Dalian 116044, China

**Keywords:** glioblastoma, resveratrol, temozolomide, STAT3, STAT3 negative regulators, MGMT

## Abstract

Chemoresistance blunts the efficacy of temozolomide (TMZ) in the treatment of glioblastoma (GBM). Elevated levels of O6-methylguanine-DNA methyltransferase (MGMT) and activation of signal transducer and of transcription 3 (STAT3) have been reported to correlate with GBM resistance to alkylator chemotherapy. Resveratrol (Res) inhibits tumor growth and improves drug chemosensitivity by targeting STAT3 signaling. Whether the combined therapy of TMZ and Res could enhance chemosensitivity against GBM cells and the underlying molecular mechanism remains to be determined. In this study, Res was found to effectively improve chemosensitivities of different GBM cells to TMZ, which was evaluated by CCK-8, flow cytometry, and cell migration assay. The combined use of Res and TMZ downregulated STAT3 activity and STAT3-regulated gene products, thus inhibited cell proliferation and migration, as well as induced apoptosis, accompanied by increased levels of its negative regulators: PIAS3, SHP1, SHP2, and SOCS3. More importantly, a combination therapy of Res and TMZ reversed TMZ resistance of LN428 cells, which could be related to decreased MGMT and STAT3 levels. Furthermore, the JAK2-specific inhibitor AG490 was used to demonstrate that a reduced MGMT level was mediated by STAT3 inactivation. Taken together, Res inhibited STAT3 signaling through modulation of PIAS3, SHP1, SHP2, and SOCS3, thereby attenuating tumor growth and increasing sensitivity to TMZ. Therefore, Res is an ideal candidate to be used in TMZ combined chemotherapy for GBM.

## 1. Introduction

Glioblastoma (GBM) is the most common primary malignant adult brain tumor and is characterized by rapid, aggressive growth, incurability, and poor prognosis [1]. Standard treatment for glioblastoma consists of surgery and radiation with concurrent temozolomide (TMZ, 75 mg/m^2^/day), followed by adjuvant 5-day TMZ therapy (150–200 mg/m^2^/day every 28 days) [2]. TMZ is an alkylating agent that induces toxicity by methylating the O^6^ position of guanine [3]. O^6^-methylguanine-DNA methyltransferase (MGMT) directly reverses the O^6^-methylguanine lesions, opposing the effect of TMZ [3]. Thus, although currently TMZ is the first-line chemotherapeutic drug for GBM, resistance to TMZ is a major problem. On the other hand, TMZ is an alkylating agent and often induces serious adverse effects, such as infertility and bone marrow suppression [4]. There is therefore an urgent need to explore more effective, less toxic treatment strategies for GBM. 

The signal transducer and activator of transcription 3 (STAT3) is widely recognized as a master regulator of the cellular functions that lead to GBM associated with poor prognosis [5]. Resveratrol (3,5,4′-trihydroxystilbene, Res), a polyphenolic compound found in red grapes and several other plants, was reported to inhibit tumor growth and improve drug chemosensitivity by targeting the STAT3 signaling pathway [6]. For example, resveratrol has been shown to overcome chemoresistance in human multiple myeloma cells through STAT3 downregulation [7]. More importantly, Res is able to cross the blood-brain barrier [8] and has little toxic effects on normal brain cells [9]. Therefore, this study aims to explore how Res combined with TMZ can improve the chemosensitivity and reduce the adverse effects of TMZ in GBM.

## 2. Results

### 2.1. Differential Chemosensitivity of GBM Cell Lines to Res and TMZ

**Figure 1:**
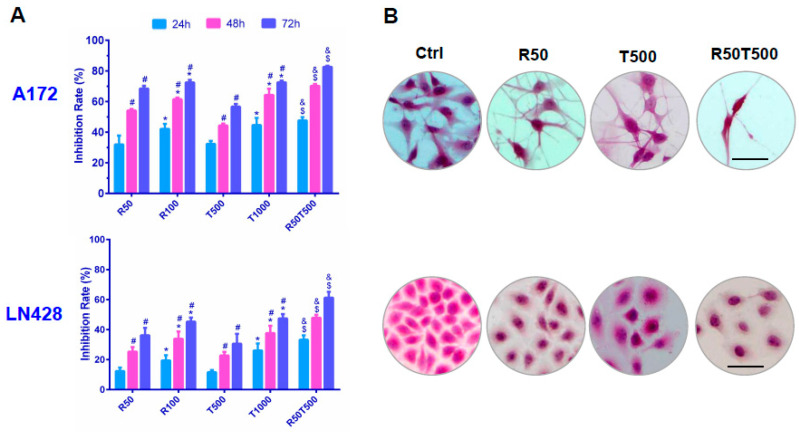
Evaluation of chemosensitivities of GBM cells to Res, TMZ, or Res + TMZ by CCK-8 (**A**) and H&E staining (**B**). All of the results represent the mean ± standard deviation of three independent experiments (*n* = 3). *, *p* < 0.05, compared with the former Res or TMZ concentration at the same time point; #, *p* < 0.05, compared with the former time point with the same Res or TMZ concentration; $, *p* < 0.05, compared with R50 or T500 at the same time point; &, *p* < 0.05, compared with T1000 at the same time point. Ctrl, control group; R50, resveratrol 50 μM; R100, resveratrol 100 μM; T500, temozolomide 500 μM; T1000, temozolomide 1000 μM; R50T500, combined treatment of resveratrol 50 μM and temozolomide 500 μM. Scale bar = 50 μm.

CCK-8 assays revealed that cell viabilities of A172 and LN428 were reduced by Res or TMZ in a dose- and time-related manner (Figure 1 and Table 1). Moreover, the two human GBM cell lines showed different chemosensitivities. The 50% inhibitory concentration (IC_50_) of Res at 48 h was 50.7 μM in A172, and 216.8 μM in LN428 cells. IC_50_ of TMZ at 48 h was 592.0 μM (A172) and 1856 μM (LN428). These data indicated that LN428 cells were much less sensitive to Res or TMZ compared with A172 cells. 

### 2.2. Res Improved Chemosensitivities of GBM Cells to TMZ

As shown in Figure 1, the growth inhibition rate of A172 treated by TMZ combined with Res was 70.2% ± 3.1% (Res 50 μM + TMZ 500 μM, 48 h), which was apparently higher than that of Res (54.2% ± 3.6%, *p* < 0.05; Res 50 μM, 48 h) or TMZ alone (44.3% ± 4.0%, *p* < 0.05, 500 μM TMZ, 48 h; even higher than 1000 μM TMZ at 48 h: 64.3% ± 4.3%, *p* < 0.05). Importantly, the inhibition rate of LN428 with the combined treatment of TMZ and Res was also much higher than TMZ alone (47.9% ± 2.4%, Res 50 μM + TMZ 500 μM, 48 h vs. 22.8% ± 3.8%, 500 μM TMZ, 48 h, *p* < 0.05; even higher than 1000 μM TMZ 48 h: 37.7% ± 2.9%, *p* < 0.05). More data are summarized in Table 1. The results demonstrate that combination therapy of TMZ and Res reduced the drug dosage of TMZ, while increasing the chemosensitivity of LN428 cells which were less sensitive to TMZ.

**Figure 2:**
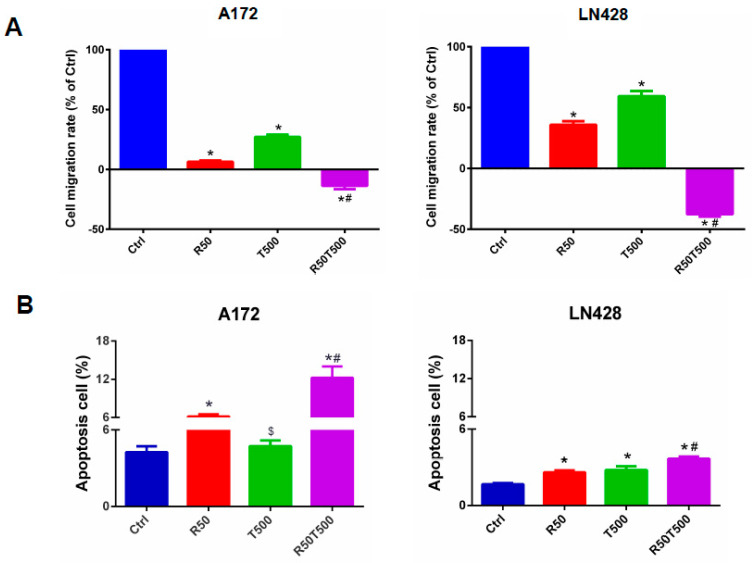
Migration and apoptosis of GBM cells treated with Res, TMZ, or Res + TMZ were checked for 48 h by in vitro scratch assays and flow cytometry. (**A**) Histograms of relative cell migration rates. Migration rate was normalized to the migration rate of the Ctrl group. (**B**) Histograms of cell apoptosis rates. All of the results represent the mean ± standard deviation of three independent experiments (*n* = 3). *, *p* < 0.01, compared with the Ctrl group; $, *p* > 0.05, compared with the Ctrl group; #, *p* < 0.01, compared with the R50 or T500. Ctrl, control group; R50, resveratrol 50 μM; T500, temozolomide 500 μM; R50T500, combined treatment of resveratrol 50 μM and temozolomide 500 μM.

In order to further investigate the anti-tumor effects of TMZ with Res combination therapy, in vitro scratch assays and flow cytometry were performed. Cell scratch tests were used to detect cell migration ability. Migration rates were normalized to the migration rates of the Ctrl group. Res, TMZ alone, or a combination of both slowed GBM cell migration (Figure 2A). After 48 h, 50 μM-Res reduced the migration rates of A172 and LN428 to 6.5% ± 1.0% and 35.8% ± 2.9% (*p* < 0.01, vs. Ctrl group), respectively. 500 μM-TMZ reduced the migration rates of A172 and LN428 to 27.1% ± 1.9% and 59.2% ± 4.3% (*p* < 0.01, vs. Ctrl group). A combination of 50 μM-Res and 500 μM-TMZ reduced the migration rates of A172 and LN428 cells to −13.6% ± 2.8% and −37.7% ± 1.9% (*p* < 0.01, vs. Ctrl group; *p* < 0.01, vs. 50 μM-Res group; *p* < 0.01, vs. 500 μM-TMZ group;) (Figure 2A). Combined treatments with Res (50 μM) and TMZ (500 μM) caused more significantly reduced migration rates than TMZ-500 μM alone. The negative migration rates mentioned above may be related to the death of GBM cells induced by Res and TMZ.

Flow cytometry assays were performed to evaluate cell apoptosis in different experimental groups. The apoptosis rates of A172 and LN428 cells increased after Res, TMZ, or a combination of both treatments for 48 h (Figure 2B, *p* < 0.05, vs. the Ctrl group). Moreover, for the two cell lines (Figure 2B), the apoptosis rates in the Res50 + TMZ500 group (A172: 12.2% ± 1.7%; LN428: 3.7% ± 0.2%) were higher than those in single drug groups (A172-Res50: 6.2% ± 0.3%, TMZ500: 4.7% ± 0.4%, *p* < 0.01; LN428-Res50: 2.6% ± 0.1%, TMZ500: 2.8% ± 0.3%, *p* < 0.01), and even higher than those in the TMZ1000 group (A172: 7.1% ± 0.4%, LN428: 3.1% ± 0.1%, *p* < 0.05). These results indicated that Res potentiated TMZ-induced GBM cell apoptosis.

### 2.3. Res Increased the Inhibitory Effects of TMZ on STAT3 Signaling in GBM Cells 

**Figure 3:**
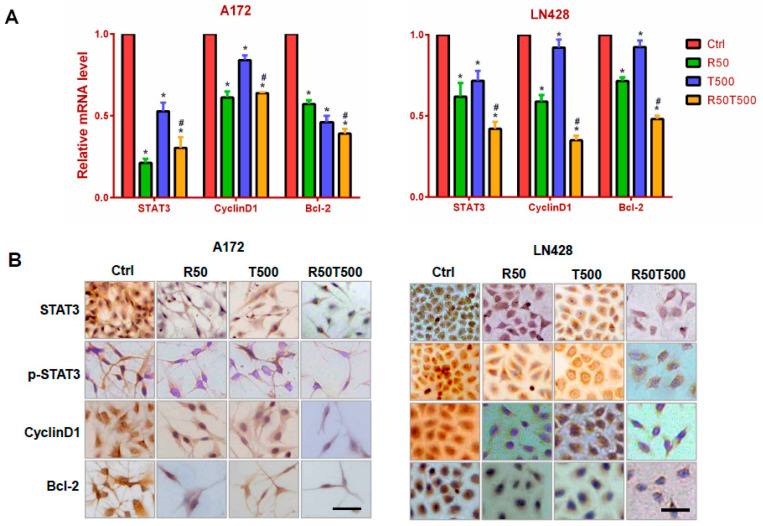
Examination of STAT3, cyclinD1, and Bcl-2 mRNA levels in A172 and LN428 cells after Res, TMZ, or Res + TMZ treatments for 48 h using qRT-PCR (**A**) and ICC (**B**). GAPDH was used as an internal control. All of the results represent the mean ± standard deviation of three independent experiments (*n* = 3). *, *p* < 0.05, compared with the Ctrl group; #, *p* < 0.05, compared with the R50 or T500. Ctrl, control group; R50, resveratrol 50 μM; T500, temozolomide 500 μM; R50T500, combined treatment of resveratrol 50 μM and temozolomide 500 μM. Scale bar = 50 μm.

Constitutive STAT3 activation has been identified in GBM and is associated with GBM development, progression and poor prognosis [10,11]. To elucidate the effects of Res or TMZ on STAT3 signaling, qRT-PCR for STAT3 was performed on RNA samples extracted from GBM cells without and with drug treatments for 48 h. As shown in Figure 3A, STAT3 was expressed in normally cultured A172 and LN428 cells, and downregulated by Res (50 μM), TMZ (500 μM), or both (*p* < 0.05, vs. the Ctrl group). Meanwhile, it was found that the decrease in STAT3 level in the TMZ combined with Res group was more obvious than that in the single TMZ group (*p* < 0.05), which reminded us that Res enhanced the inhibitory effects of TMZ on STAT3 in GBM cells. ICC staining was performed on the same experimental groups to further check the location and relative abundance of STAT3 and p-STAT3. The results showed that STAT3 was distributed in the cytoplasm and nuclei of A172 and LN428 cells under normal culture conditions, which were reduced after Res or TMZ treatment, especially in the combined treated cells (Figure 3B and Supplementary Figure S1). ICC staining for p-STAT3 showed positive staining mainly in the nuclei of A172 and LN428. After Res, TMZ, or combined treatments, p-STAT3 immunolabeling was diminished in the nuclei (Figure 3B and Supplementary Figure S1). Moreover, the decrease of STAT3 or p-STAT3 in LN428 treated with TMZ alone was not as obvious as that in A72 cells. After combined treatments of TMZ and Res, either STAT3, or p-STAT3 was obviously reduced in LN428 cells.

To further explore the effects of Res, TMZ, or both on STAT3 downstream genes, levels of Cyclin D1 and Bcl-2 were examined by qRT-PCR and ICC staining. The results showed that Cyclin D1 was expressed in normally cultured A172 and LN428 cells, and downregulated (Figure 3 and Supplementary Figure S1, *p* < 0.05, vs. the Ctrl group) after 50 μM-Res or 500 μM-TMZ treatment, especially the combined treatment of TMZ and Res (Figure 3 and Supplementary Figure S1, *p* < 0.05, vs. TMZ500). The similar pattern of Bcl-2 expression was also evidenced in the two cell lines (Figure 3 and Supplementary Figure S1, *p* < 0.05, vs. the Ctrl group; TMZ500 μM vs. Res50 μM + TMZ500 μM, *p* < 0.05). The results suggest that Res can enhance the inhibitory effects of TMZ on STAT3 signaling in GBM cells.

### 2.4. Res and TMZ Upregulated PIAS3, SOCS3, SHP1, and SHP2 Levels in GBM Cells

**Figure 4:**
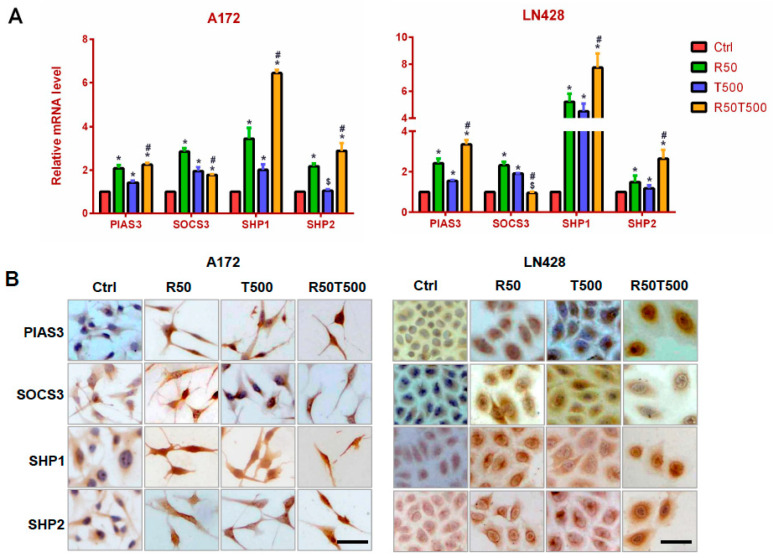
Evaluation of PIAS3, SOCS3, SHP1, and SHP2 levels in A172 and LN428 cells after Res, TMZ, or Res + TMZ treatments for 48 h using qRT-PCR (**A**) and ICC (**B**). GAPDH was used as an internal control. All of the results represent the mean ± standard deviation of three independent experiments (*n* = 3). *, *p* < 0.05, compared with the Ctrl group; $, *p* > 0.05, compared with the Ctrl group; #, *p* < 0.05, compared with the R50 or T500. Ctrl, control group; R50, resveratrol 50 μM; T500, temozolomide 500 μM; R50T500, combined treatment of resveratrol 50 μM and temozolomide 500 μM. Scale bar = 50 μm.

PIAS3, SOCS3, SHP1, and SHP2 are known negative regulators of STAT3 signaling [12,13,14,15], and were examined to explore the molecular mechanism for inhibited STAT3 signaling in GBM cells treated with Res and TMZ. The qRT-PCR results showed that PIAS3, SHP1, and SHP2 were upregulated by 50 μM-Res, 500 μM-TMZ, or both in A172 and LN428 cells, especially SHP1 (Figure 4A, *p* < 0.05, vs. the Ctrl group). Furthermore, upregulation of PIAS3, SHP1, and SHP2 in the TMZ combined with Res groups was more obvious than that in the single drug groups (Figure 4A, *p* < 0.05, vs. 50 μM-Res or 500 μM-TMZ groups). For SOCS3, its expression pattern was different. SOCS3 was also upregulated by 50 μM-Res, 500 μM-TMZ, or both in A172, but its upregulated level in the TMZ500-combined-with-Res50 group was less obvious than that in the single drug group (Figure 4A, *p* < 0.05, vs. 50 μM-Res or 500 μM-TMZ group). In LN428 cells, SOCS3 was only upregulated by TMZ or Res alone (Figure 4A, *p* < 0.05, vs. the Ctrl group), and not obviously changed in LN428 after TMZ combined with Res treatment (*p* = 0.082, vs. the Ctrl group). 

ICC staining was used to further check the location and relative intensity of PIAS3, SOCS3, SHP1, and SHP2 in GBM cells. The results showed that PIAS3, SHP1, and SHP2 were distributed mainly in the cytoplasm of A172 and LN428 cells under normal culture conditions, which became increased in the cytoplasm and nuclei after Res, TMZ alone, or both treatments (Figure 4B and Supplementary Figure S2). ICC staining for SOCS3 showed positive staining mainly in the cytoplasm of A172 and LN428 cells. Its upregulation occurred mainly in tumor cells treated with a single drug (Res 50 μM or TMZ 500 μM). After the combination of 50 μM-Res and 500 μM-TMZ treatment, SOCS3 levels were different in the two cell lines: upregulated in A172 cells, downregulated in LN428 cells (Figure 4B and Supplementary Figure S2). 

### 2.5. The Effects of Res on MGMT Level and Its Relevance with Different Chemosensitivities of GBM Cells to TMZ

**Figure 5:**
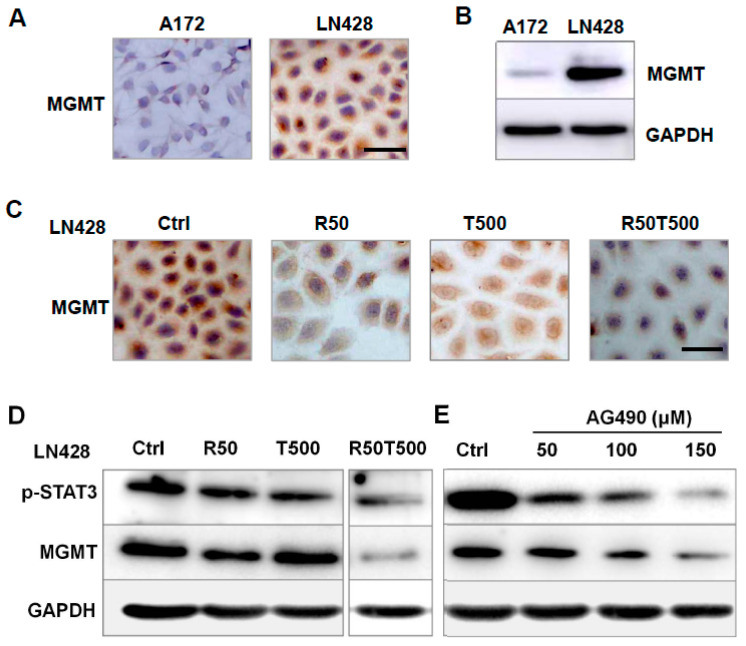
Analyses of MGMT expression pattern in A172 and LN428 cells with different treatments. (**A**,**B**) Examination of MGMT level in A172 and LN428 cells by ICC (**A**) and western blotting (**B**). (**C**) Effects of Res 50 μM, TMZ 500 μM, or both on MGMT levels in LN428 cells by ICC. (**D**) Effects of Res 50 μM, TMZ 500 μM, or both on p-STAT3 and MGMT levels in LN428 cells by western blotting. (**E**) Effects of AG490 on p-STAT3 and MGMT protein levels in LN428 cells. GAPDH was used as an internal control. All of the results represent the mean ± standard deviation of three independent experiments (*n* = 3). Ctrl, control group; R50, resveratrol 50 μM; T500, temozolomide 500 μM; R50T500, combined treatment of resveratrol 50 μM and temozolomide 500 μM. Scale bar = 50 μm.

MGMT is involved in TMZ resistance in GBM cells [16,17]. Thus, MGMT was investigated to evaluate its relevance with chemosensitivity of GBM cells to TMZ. As shown in Figure 5A,B, LN428 cells expressed a higher level of MGMT protein compared with A172, which may be the main reason for the different chemosensitivities of GBM cells to TMZ: LN428 was less sensitive than A172 to TMZ (Supplementary Figure S3). 

So the effects of Res and TMZ on MGMT level were further checked in LN428 cells. ICC staining showed positive staining mainly in the cytoplasm of LN428 cells (Figure 5C and Supplementary Figure S3). After Res alone or combined Res with TMZ treatments, MGMT immunolabeling was apparently diminished, but did not obviously change after a single 500 μM-TMZ treatment (Figure 5C and Supplementary Figure S3). The results revealed that TMZ alone imposed very little effect on MGMT level, but TMZ combined with Res could effectively decrease MGMT level, which may be the reason why TMZ combined with Res reversed the chemoresistance of LN428 to TMZ.

### 2.6. AG490 Downregulated MGMT Level in LN428 Cells

As shown in Figure 5D, after combined Res with TMZ treatments, the protein levels of p-STAT3 and MGMT were apparently diminished in LN428 cells. To determine the correlation between the status of STAT3 activation and MGMT level, AG490, a selective inhibitor of STAT3 phosphorylation [18], was used to treat LN428 cells. A western blot analysis demonstrated that p-STAT3 levels in LN428 cells decreased differently by 50 μM, 100 μM, or 150 μM AG490 in a dose-dependent fashion (Figure 5E). Meanwhile, the MGMT level was also downregulated by AG490 in a dose-related fashion (Figure 5E), suggesting that the MGMT level was downregulated by STAT3 inactivation.

## 3. Discussion

The current standard treatment for GBM includes surgery followed by radiotherapy and chemotherapy [2]. TMZ, as a first-line chemotherapeutic drug, frequently fails to prevent the development of resistance and fatal recurrences. There is an urgent need to develop high-efficiency and low-toxicity strategies for GBM. Res has good anticancer activity and can pass through the blood-brain barrier [8,9], thus a combination strategy of TMZ and Res was developed in this study to determine whether a combination therapy of TMZ with Res could improve the chemosensitivity of GBM cells to TMZ, and thereby reduce TMZ dosage and its adverse effects.

In order to investigate the efficacy of the combination therapy strategy in overcoming drug resistance and the serious side effects of TMZ in GBM, different chemosensitivities of GBM cells to TMZ were first checked, and it was found that A172 cells were more sensitive than LN428 (less sensitive). Furthermore, compared with Res or TMZ alone, TMZ combined with Res greatly enhanced the anti-tumor efficacy of TMZ on A172 cells. Res is known to cross the blood-brain barrier [8,19] and have low toxic effects, which means that the combination treatment (TMZ + Res) can not only improve the anti-tumor effects of TMZ, but also decreases the TMZ dose, thereby alleviating its side effects. Notably, the use of TMZ together with Res made LN428 cells more sensitive to TMZ, providing new hope for patients who are clinically TMZ resistant (primary or recurrent).

Aberrant activation of STAT3 has been found in GBM [20,21], and correlates with mesenchymal differentiation and poor prognosis in human gliomas [22]. Res is reported to exert its anti-tumor effects mainly by inhibiting STAT3 signaling [7,23,24]. Therefore, the effects of Res and TMZ on STAT3 signaling were investigated in GBM cells. In A172 cells (sensitive to TMZ), the activity of STAT3 signaling was obviously suppressed by TMZ alone, but particularly when combined with Res. However, for LN428 cells (less sensitive to TMZ), STAT3 signaling was not suppressed as obviously as in A172 cells after single TMZ treatment, which may be one reason that LN428 cells were less sensitive to TMZ than A172 cells. This is consistent with STAT3 being a therapeutic target for TMZ resistance in GBM [25,26]. Moreover, after TMZ was combined with Res, the activity of STAT3 signaling was significantly reduced, along with the increased sensitivity of LN428 to TMZ. This further confirmed the correlation between STAT3 level and TMZ resistance. Temozolomide induces DNA methylation of guanine at the O^6^ position and triggers the mismatch repair (MMR) system leading to a DNA double strand break that results in cell cycle arrest and apoptosis [27,28]. Nonetheless, in this study another anti-tumor molecular mechanism of TMZ was revealed: preventing cell growth by inhibiting STAT3 signaling.

STAT3 signaling is negatively regulated by PIAS3, SOCS3, SHP1, and SHP2 [12,29,30,31]. According to our findings, PIAS3, SHP1, and SHP2 were obviously upregulated by Res, TMZ, or both in GBM cells. Moreover, upregulation of PIAS3, SHP1, and SHP2 in TMZ combined with Res groups was more obvious than that in a single drug group. It is strongly suggested that the inhibitory effects of TMZ, Res, or both on STAT3 signaling may be related to the upregulation of these negative regulators. It is reported that reduced PIAS3 in glioblastoma tissues promotes tumor-cell proliferation, and PIAS3 overexpression inhibits glioblastoma-cell growth by reducing STAT3 [32,33]. The tumor suppressive activities of SHP1 and SHP2 occur mainly through their inactivation of STAT3. Verbascoside is proven to inhibit glioblastoma-cell proliferation, migration, and invasion through upregulation of SHP-1 and inhibition of STAT3 phosphorylation [34]. Studies have also revealed that SHP2-deficient mice present increased STAT3 activity and increased hepatocellular adenomas [35]. These are consistent with our study. 

However, SOCS3 is different. Besides being a negative regulator, SOCS3 is the target gene of STAT3 signaling, thus forming a negative feedback loop between SOCS3 and STAT3 [36]. The SOCS3 level showed an increase mainly in the single Res or TMZ group. Although in the TMZ combined with Res group, its level was not obviously changed in LN428. It is suggested that changes in SOCS3 level may be a consequence of the negative regulatory action of PIAS3, SHP1, and SHP2 on STAT3 signaling and negative feedback between STAT3 signaling and SOCS3. 

A number of cases have been reported to be resistant to TMZ due to elevated MGMT [37]. In this study, MGMT level was checked, and it was found that LN428 cells exactly expressed a higher level of MGMT protein compared with A172 cells, which is consistent with the lower sensitivity of LN428 cells to TMZ. Notably, the TMZ combined with Res treatment reversed the decreased sensitivity of LN428 to TMZ. To further confirm the correlation between MGMT and TMZ resistance, we checked MGMT and found that TMZ imposed little effect on MGMT, and the TMZ combined with Res treatment significantly reduced MGMT levels. Thus, TMZ combined with Res increases the chemosensitivity of LN428 to TMZ possibly by downregulating the MGMT level, too.

In addition, our study shows a regulatory role of STAT3 for the MGMT level. In LN428 cells, a decreased MGMT level was accompanied by downregulation of p-STAT3. To ensure possible p-STAT3-mediated regulation on MGMT, AG490 was used to treat LN428 cells. P-STAT3 level was decreased by AG490 in a dose-dependent fashion. Meanwhile, MGMT level was also downregulated by AG490 in a dose-related fashion, suggesting that downregulated MGMT level was related to STAT3 inactivation. A positive correlation between MGMT level and phosphorylated STAT3 has been reported in human malignant gliomas [38], which is consistent with our report. Further analysis should be required for the p-STAT3-regulated-MGMT mechanism. Therefore, it is strongly suggested that STAT3 is a therapeutic target for TMZ resistance in GBM. 

**Figure 6:**
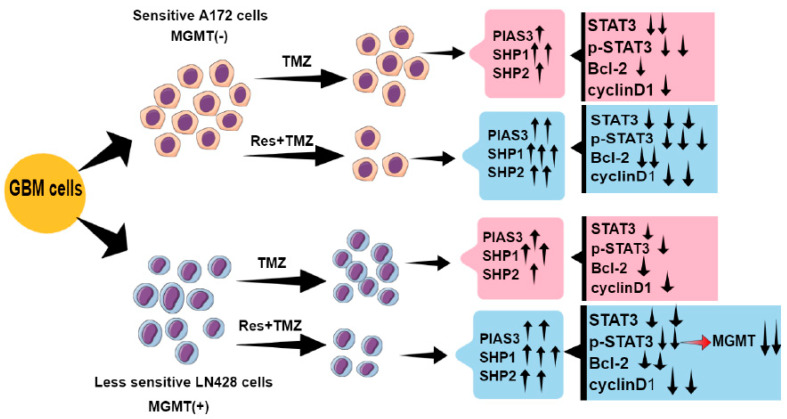
Schematic illustration of this study. ↑: increase; ↓: decrease.

In conclusion, the TMZ combined with Res therapy strategy is able to increase the chemosensitivities of GBM cells to TMZ, reduce TMZ dose, and thereby alleviate its adverse effects (Figure 6). Res and TMZ exert the anti-tumor effects mainly by inhibiting STAT3 signaling, and inhibition of STAT3 signaling might be related to upregulation of PIAS3, SHP1, SHP2, and SOCS3 (Figure 6). Additionally, we show a regulatory role of p-STAT3 on MGMT level: TMZ combined with Res reduces the MGMT level mediated by STAT3 inactivation (Figure 6). Therefore, a STAT3 inhibitor could be one of the candidate drugs for TMZ resistant GBM patients. In our study, Res has proven to be a drug which enhances TMZ efficacy and reverses TMZ resistance by deactivating STAT3 signaling. Moreover, Res has known advantages: it easily crosses the blood-brain barrier and has fewer toxic effects on normal brain cells. All of these characteristics make Res viable to be used in GBM’s combined chemotherapy.

## 4. Materials and Methods

### 4.1. Cell Culture and Treatments

Human GBM cell lines A172 and LN428 were purchased from the Type Culture Collection of the Chinese Academy of Sciences (Shanghai, China) and cultured in Dulbecco’s modified Eagle’s essential medium (DMEM) containing 10% fetal bovine serum (Gibco Life Science, Grand Island, NY, USA) under 37 °C and 5% CO_2_ conditions. 

TMZ and Res were purchased from Sigma-Aldrich (St. Louis, MO, USA) and dissolved in dimethylsulfoxide (DMSO; Sigma-Aldrich, Darmstadt, Germany) and diluted before use. The cells under normal culture conditions, treated by 0.2% DMSO were used as normal and background controls. GBM cells were treated with 10 μM, 25 μM, 50 μM, and 100 μM Res; 100 μM, 250 μM, 500 μM, 750 μM, and 1000 μM TMZ; and 50 μM Res combined with 500 μM TMZ and 100 μM Res combined with 500 μM for 72 h, respectively. Cell numbers and viabilities were checked at 24 h intervals. The cell-bearing coverslips were fixed in cold acetone or 4% paraformaldehyde (pH 7.4) for morphological and immunocytochemical examinations. The experimental groups were set in triplicate and the experiments were repeated three times to establish confidential conclusion.

### 4.2. Cell Proliferation Assays

The effects of Res, TMZ alone, and Res combined with TMZ on GBM cell proliferation were determined by CCK-8 (Cell Counting Kit-8, Sigma-Aldrich Co., USA) assay. 1 × 10^4^ cells/mL were seeded in 96-well plates and absorbance was read at 450 nm at 24, 48, and 72 h, respectively, after being incubated with CCK-8 for 2 h. The results were obtained as a percentage of cell growth inhibition [(1-OD of the experiment samples/OD of the controls) × 100%]. Hematoxylin and eosin (H/E) staining was performed to observe the morphological changes of GBM cells after treatments.

### 4.3. Flow Cytometry Analysis

The apoptosis assay was performed in cells using Annexin V-FITC Apoptosis Detection Kit (Beyotime Institute of Biotechnology, Shanghai, China) and assessed following flow on an LSR III (BD Biosciences, San Jose, CA, USA) and FlowJo (FlowJo LLC, Ashland, OR, USA) analysis software.

### 4.4. Cell Migration Assays

To detect the effects of Res, TMZ alone, and their combination on GBM cell migration, in vitro scratch assays were performed. Sterile plastic 200-μL micropipette tips were used to scratch a layer of confluent cells and thus create a linear wound. The scratched cells were then washed with serum-free media, and the medium was changed to DMEM with 2% FBS and treated with different concentrations of Res, TMZ alone, and their combination. Micrographs of the scratches were taken at 0 h, immediately after the scratch, and at 48 h after drug treatments. The wound closure area was measured using the software Image J and normalized to the wound closure area in the control group.

### 4.5. RNA Isolation and qRT-PCR

Total cellular RNA of each experimental group was extracted using Trizol solution (Life Tech, Houston, TX, USA). For quantitative real-time PCR, RNA samples (1 μg) were reversely transcribed in a final volume of 20 μL containing Prime Script RT reagents (TaKaRa) according to the manufacturer’s protocol to generate cDNA. Real-time quantitative PCR was conducted following the protocol supplied with the SYBR^®^premix Dimer Eraser^TM^ kit (TaKaRa). Gene specific primers used in this study were designed, synthesized by Takara Bio Inc and are listed in Table 2. The relative expression of target mRNA was determined using the comparative threshold (Ct) method by normalizing target mRNA Ct values to those for GAPDH (ΔCt). The following formula was used: ΔΔCT = ΔCT sample − ΔCT calibrator. This value was used to plot the gene expression employing the formula: 2^−ΔΔCT^.

### 4.6. Immunocytochemical (ICC) Staining 

ICC was performed on the coverslips obtained from each of the experimental groups by the method described previously [23]. STAT3 (1:1000), cyclinD1 (1:200), Bcl-2 (1:500), SOCS3 (1:200), PIAS3 (1:200), SHP1 (1:300), and SHP2 (1:300) antibodies were purchased from Proteintech Group, Inc. (Chicago, USA), MGMT (1:800) and p-STAT3 (Tyr705, 1:200) from Santa Cruz Biotechnology, Inc., CA, USA and used according to the manufacturer’s instruction. 

### 4.7. Western Blotting

Total cellular proteins were prepared according to the method previously described [39]. Expression of MGMT and p-STAT3 (phosphorylation at Tyr705) was determined using western blot analysis. MGMT (mouse monoclonal antibody, 1:500) and p-STAT3 (mouse monoclonal antibody, 1:1000) antibodies were purchased from Santa Cruz Biotechnology, USA. Meanwhile, an antibody against GAPDH (ProteinTech Group Inc., Chicago, USA, 1:10,000) was used to stain GAPDH as a loading control.

### 4.8. Inhibition of STAT3 Activation with AG490

JAK2-specific inhibitor AG490 (Sigma) [40] was dissolved in DMSO and diluted to the working concentrations of 50 μM, 100 μM, and 150 μM with a culture medium just before use. Five experimental groups were set as follows: Group 1, normal culture; Group 2, treatment with 0.2% DMSO as background control; Groups 3, 4, and 5 treatment with 50 μM, 100 μM, or 150 μM AG490. The effects of AG490 on p-STAT3 and MGMT expressions in LN428 cells were determined by western blotting.

### 4.9. Statistical Analysis

Statistical analysis was conducted using SPSS 21.0 (IBM Corp., Armonk, NY, USA). Data are presented as the mean ± standard deviation. Comparisons between two groups were analyzed using t-tests. Comparisons among multiple groups were assessed using analysis of variance. Statistical significance was defined as *p* < 0.05.

## Figures and Tables

**Table 1:** Growth inhibition rates (%) of Res and TMZ on GBM cells.

	**GBM A172 Cells**										
**Treatment**	**Res (μM)**				**TMZ (μM)**					**Res (μM) + TMZ (μM)**	
**Time (h)**	**10**	**25**	**50**	**100**	**100**	**250**	**500**	**750**	**1000**	**50 + 500**	**100 + 500**
24	9.1 ± 0.5	16.7 ± 1.2 *	32.0 ± 2.8 *	42.2 ± 2.6 *	11.7 ± 0.8	21.4 ± 1.4 *	32.3 ± 2.9 *	37.8 ± 4.0 *	44.7 ± 3.8	47.7 ± 2.2 ^^&^	55.5 ± 2.9 ^^&^
48	22.4 ± 2.0 ^#^	32.4 ± 2.8 *^#^	54.2 ± 3.6 *^#^	61.5 ± 3.6 *^#^	22.1 ± 1.8 ^#^	30.9 ± 2.2 *^#^	44.3 ± 4.0 *^#^	53.3 ± 3.6 *^#^	64.3 ± 4.3 *^#^	70.2 ± 3.1 ^^&^	76.9 ± 4.6 ^^&^
72	24.8 ± 2.6	35.7 ± 3.1 *	68.4 ± 4.7 *^#^	72.5 ± 4.1 *^#^	26.5 ± 2.4	37.7 ± 2.7 *^#^	56.6 ± 4.8 *^#^	69.7 ± 5.1 *^#^	72.5 ± 4.4 *^#^	82.6 ± 5.0 ^^&^	84.1 ± 5.0 ^^&^
	**GBM LN428 cells**										
**Treatment**	**Res (μM)**				**TMZ (μM)**					**Res (μM) + TMZ (μM)**	
**Time (h)**	**10**	**25**	**50**	**100**	**100**	**250**	**500**	**750**	**1000**	**50 + 500**	**100 + 500**
24	1.2 ± 0.5	6.9 ± 0.7 *	12.4 ± 1.0 *	19.5 ± 2.8 *	3.9 ± 0.6	5.5 ± 1.2 *	11.6 ± 0.9 *	15.6 ± 3.0 *	26.1 ± 3.0 *	33.2 ± 1.2 ^^&^	33.7 ± 3.1 ^^&^
48	4.7 ± 0.6 ^#^	17.5 ± 1.3 *^#^	25.3 ± 1.5 *^#^	33.9 ± 1.2 *^#^	7.5 ± 0.5 ^#^	13.8 ± 2.3 *^#^	22.8 ± 3.8 *^#^	28.9 ± 2.9 *^#^	37.7 ± 2.9 *^#^	47.9 ± 2.4 ^^&^	53.5 ± 4.2 ^^&^
72	10.0 ± 1.1 ^#^	29.6 ± 1.4 *^#^	36.2 ± 2.0 *^#^	45.3 ± 2.7 *^#^	20.3 ± 1.2 ^#^	25.6 ± 2.8 *^#^	30.6 ± 3.1 *^#^	37.7 ± 3.2 *^#^	47.5 ± 4.1 *^#^	61.3 ± 3.8 ^^&^	67.8 ± 4.6 ^^&^

All of the results represent the mean ± standard deviation of three independent experiments (*n* = 3); Res: resveratrol, TMZ: temozolomide. *, *p* < 0.05, compared with the former Res or TMZ concentration at the same time point; ^#^, *p* < 0.05, compared with the former time point with the same Res or TMZ concentration; ^^^, *p* < 0.05, compared with T500 at the same time point; ^&^, *p* < 0.05, compared with T1000 at the same time point.

**Table 2:** Sequences of RT—real-time PCR primers.

Parameters	Primer Sequences	Product Size (bp)
STAT3	F: 5′-TTCACTTGGGTGGAGAAGGACA-3′ R: 5′-CGGACTGGATCTGGGTCTTACC-3′	49
PIAS3	F: 5′-ACTACATGAGTACCCACCTGCCTTC-3′ R: 5′-CCAAGGGCATCCTGTTCATCTA-3′	135
SOCS3	F: 5′-CAGGAATGTAGCAGCGATGGAA-3′ R: 5′-CCTGTCCAGCCCAATACCTGA-3′	125
SHP1	F: 5′-GGGATTTCTATGACCTGTATGGAG-3′ R: 5′-CCAGACATGTGGCCATGGTA-3′	178
SHP2	F: 5′-AAGAATATGGCGTCATGCGTGTTA-3′ R: 5′-GCCAGGTCCGAAAGTGGTATTG-3′	150
Bcl-2	F: 5′-CCCGTTGCTTTTCCTCTGG-3′ R: 5′-ATCCCACTCGTAGCCCCTCT-3′	117
CyclinD1	F: 5′-TGTTCGTGGCCTCTAAGATGAA-3′ R: 5′-TCGGTGTAGATGCACAGCTTCT-3′	67
GAPDH	F: 5′-GCACCGTCAAGGCTGAGAAC-3′ R: 5′-TGGTGAAGACGCCAGTGGA-3′	138

## Data Availability

Not applicable.

## Supplementary Materials

The following supporting information can be downloaded at: https://www.mdpi.com/article/10.3390/ijms24119453/s1.
