# Supplementary material for: Resveratrol Enhances Temozolomide Efficacy in Glioblastoma Cells through Downregulated MGMT and Negative Regulators-Related STAT3 Inactivation
Source: Int J Mol Sci. 2023 May 29;24(11):9453. doi: 10.3390/ijms24119453 (PMC10253519; doi:10.3390/ijms24119453)
Supplement: Supplementary file 1 [file ijms-24-09453-s001.zip › Supplementary file/Supplementary Figure S1.pdf]

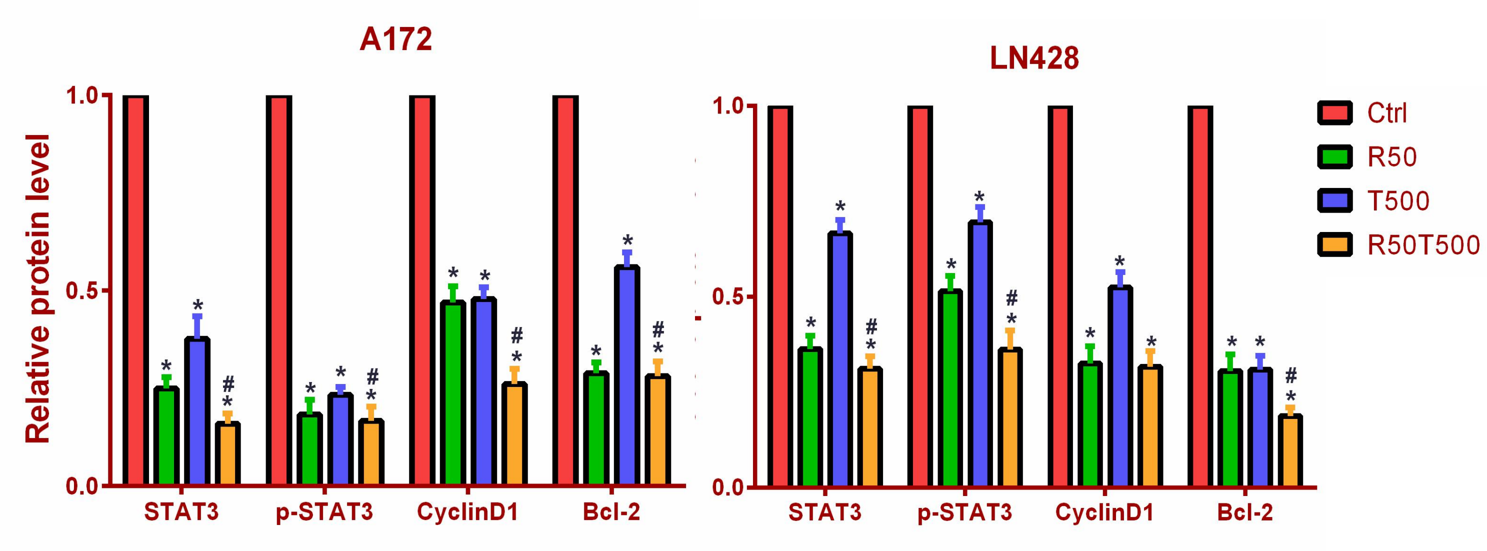

Supplementary Fig. S1. Quantiative analyses of STAT3, p-STAT3, CyclinD1 and Bcl-2 levels in A172 and LN428 cells by ICC (shown in Fig. 3B). The data represent the mean ± standard deviation of three independent experiments (*n* = 3). *, *p*<0.05, compared with the Ctrl group; #, *p*<0.05, compared with the R50 or T500. Ctrl, control group; R50, resveratrol 50μM; T500, temozolomide 500μM; R50T500, combine treatment of resveratrol 50μM and temozolomide 500μM.
