# Supplementary material for: Resveratrol Enhances Temozolomide Efficacy in Glioblastoma Cells through Downregulated MGMT and Negative Regulators-Related STAT3 Inactivation
Source: Int J Mol Sci. 2023 May 29;24(11):9453. doi: 10.3390/ijms24119453 (PMC10253519; doi:10.3390/ijms24119453)
Supplement: Supplementary file 1 [file ijms-24-09453-s001.zip › Supplementary file/Supplementary Figure S3.pdf]

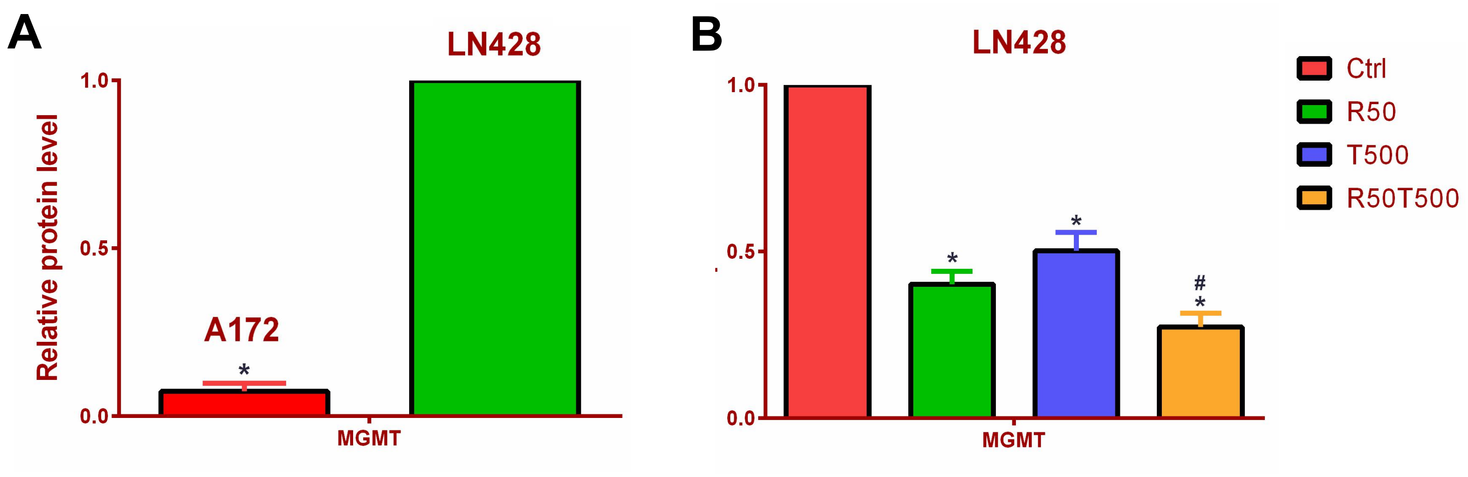

Supplementary Fig. S3. Quantiative analyses of MGMT levels in A172 and LN428 cells by ICC. (A) Histograms of MGMT levels in A172 and LN428 cells by ICC (shown in Fig. 5A). (B) Histograms of MGMT levels in LN428 cells with different treatments by ICC (shown in Fig. 5C). The data represent the mean ± standard deviation of three independent experiments (*n* = 3). *, $p<0.05$, compared with the Ctrl group; #, $p<0.05$, compared with the R50 or T500. Ctrl, control group; R50, resveratrol 50μM; T500, temozolomide 500μM; R50T500, combine treatment of resveratrol 50μM and temozolomide 500μM.
